# Common bile duct villous adenoma: a case report and review of the literature

**DOI:** 10.1186/s13256-015-0789-z

**Published:** 2016-01-21

**Authors:** Karolis Čekas, Vilius Rudaitis, Virgilijus Beiša, Valdemaras Jotautas, Dileta Rutkauskaitė, Raimundas Meškauskas, Eugenijus Stratilatovas

**Affiliations:** 1 Faculty of Medicine, Vilnius University, M. K. Čiurlionio Street 21, 03101 Vilnius, Lithuania; 2 Clinic of Internal Diseases, Family Medicine and Oncology, Faculty of Medicine, Vilnius University, Department of Gynecology, Center of Women’s Physiology and Pathology, Vilnius University Hospital Santariškių Klinikos, Santariškių Street 2, 08661 Vilnius, Lithuania; 3 Clinic of Gastroenterology, Nephrourology and Surgery, Faculty of Medicine, Vilnius University, Center of Abdominal Surgery, Vilnius University Hospital Santariškių Klinikos, Santariškių Street 2, 08661 Vilnius, Lithuania; 4 Department of Radiology, Nuclear Medicine and Medical Physics, Faculty of Medicine, Vilnius University, Center of Radiology and Nuclear Medicine, Vilnius University Hospital Santariškių Klinikos, Santariškių Street 2, 08661 Vilnius, Lithuania; 5 National Center of Pathology, P. Baublio 5, 08406 Vilnius, Lithuania

**Keywords:** Obstructive jaundice, Benign tumor, Extrahepatic bile ducts, Roux-en-Y hepaticojejunostomy

## Abstract

**Background:**

According to the literature, benign bile duct tumors are exceedingly uncommon. To the best of our knowledge, we report the largest extrahepatic bile duct villous adenoma described in the literature.

**Case presentation:**

We present a case of a 77-year-old Caucasian woman with obstructive jaundice. Laboratory tests revealed that she had elevated bilirubin and liver enzyme levels. A computed tomographic scan showed a homogeneous 5 × 3–cm mass obstructing the common bile duct. The results of brush cytology were consistent with a bile duct villous papilloma. However, on the basis of the tumor’s radiological features, a preliminary diagnosis of extrahepatic bile duct malignant tumor was made. After discussion among the multidisciplinary team, a surgical resection of the bile duct tumor was performed. Histopathological examination confirmed a villous adenoma. The patient’s postoperative course was uneventful.

**Conclusions:**

In patients with bulky extrahepatic bile duct tumors, surgical resection alone may be safe and curative.

## Background

An extrahepatic bile duct villous adenoma is an exceedingly rare benign epithelial tumor [1, 2]. We present a case of a patient with a bulky villous adenoma of the common bile duct that was diagnosed and successfully treated at Vilnius University Hospital Santariškių Klinikos in Lithuania.

## Case presentation

A 77-year-old Caucasian woman was admitted to Panevėžys regional hospital in Lithuania because of a 1-day history of skin and scleral jaundice and weakness. Her medical history included a laparoscopic cholecystectomy 15 years earlier for a gallbladder stone.

A clinical examination revealed only skin and scleral jaundice. The patient’s laboratory test results were as follows: total bilirubin (TBIL) 338.59 U/L (normal <21 U/L), direct (conjugated) bilirubin (DBIL) 294 U/L (normal <5.3), alkaline phosphatase (ALP) 720.1 U/L (normal 40–129), alanine transaminase (ALT) 52.0 U/L (≤35), aspartate transaminase (AST) 142.1 U/L (normal ≤35), γ-glutamyl transpeptidase (GGT) 353.0 U/L (normal 9–40), and carbohydrate antigen 19-9 157.09 U/ml (normal 0–37). On the basis of ultrasound and computed tomography (CT) findings, cancer in the head of the pancreas with invasion to the common bile duct (CBD) was suspected. To alleviate jaundice and to perform brush cytology, percutaneous transhepatic bile duct drainage was performed. After this procedure, decreases in TBIL (187.92 U/L), DBIL (95.69 U/L), ALP (276.1 U/L), ALT (37.2 U/L), AST (73.0 U/L), and GGT (123.8 U/L) were noted.

**Fig. 1:**
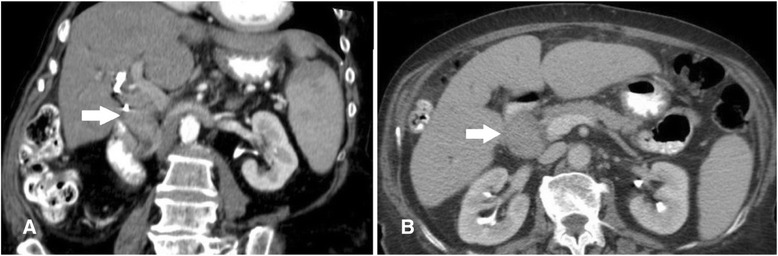
Computed tomographic scan shows the presence of common bile duct–obstructing homogeneous mass (*arrows*). **a** Coronal view. **b** Axial view

A bile duct villous papilloma was suspected on the basis of brush cytology. Despite the results of these investigations, a chance of malignancy remained. The patient was referred to the local tertiary treatment center for further treatment. At the Vilnius University Hospital Santariškių Klinikos, a multidisciplinary team reviewed a CT scan of the patient. The scan showed the presence of a CBD-obstructing, homogeneous, 5 × 3–cm mass below the fusion of extrahepatic ducts without any evidence of tumor metastasis in a common bile duct surrounding tissue, the liver, right and left intrahepatic ducts, and the pancreas. The patient’s periportal lymph nodes were of normal size. On the basis of the tumor’s radiological features, a preoperative diagnosis of extrahepatic bile duct malignant tumor was made (T1N0M0, type I, by Bismuth-Corlette classification) (Fig. 1).

The multidisciplinary team assessed the absence of tumor invasion in surrounding tissues and decided to perform a surgical resection of the tumor. However, there remained a possibility to perform a more radical operation, such as pancreaticoduodenectomy, if the cancer was observed to involve the distal part of the CBD.

A laparotomy was performed. The duodenum and the head of the pancreas were mobilized by using the Kocher maneuver. Importantly, no trace of tumor was found in the proximal common hepatic duct (CHD) and distal CBD parts. Only bile duct parts containing tumor were resected. To perform a Roux-en-Y hepaticojejunostomy, a small intestine loop was mobilized and divided 50 cm from the superior duodenal fold. The distal part was closed and anastomosed end to side to the remaining 0.5-cm length of the CHD. Sixty centimeters below this junction, an end-to-side jejunojejunal anastomosis was formed.

**Fig. 2:**
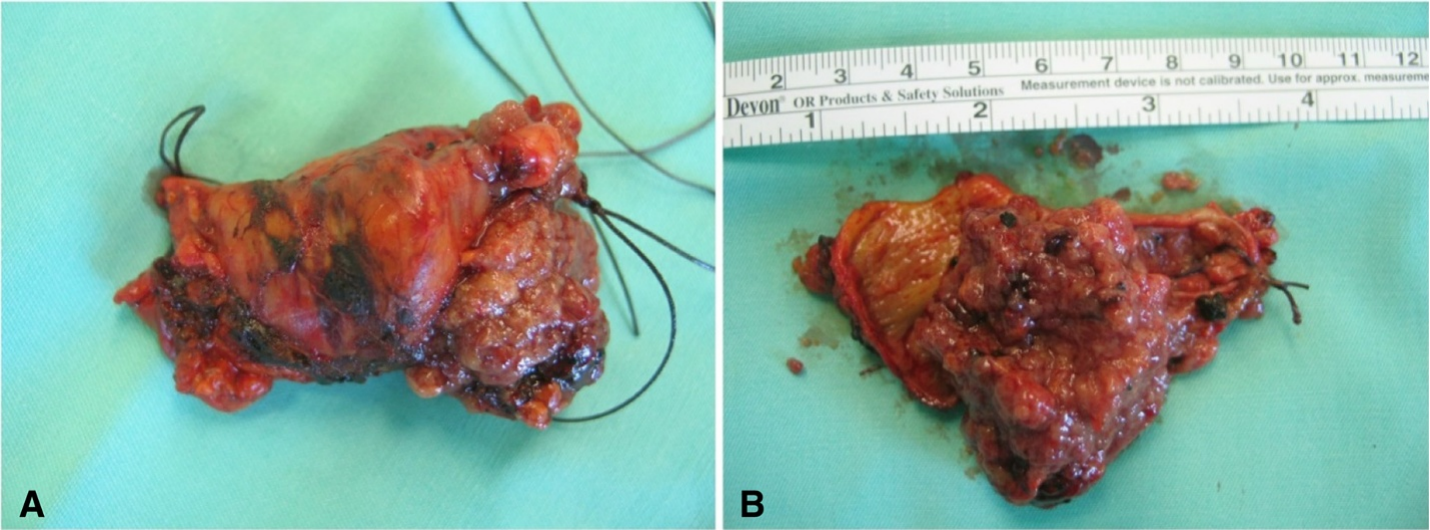
Surgical-pathological findings of a tumor inside the bile duct. **a** Intact bile duct. **b** Dissected bile duct

Macroscopic evaluation of the specimen revealed that the resected parts of the CBD and CHD consisted of dilated bile ducts with a papillary tumor protruding into the lumen. The tumor’s size was 4.5 × 4 × 2.5 cm. The resection margin was 2 cm from one side and 2.5 cm from the other, and two investigated lymph nodes were identified as tumor-free (Fig. 2).

**Fig. 3:**
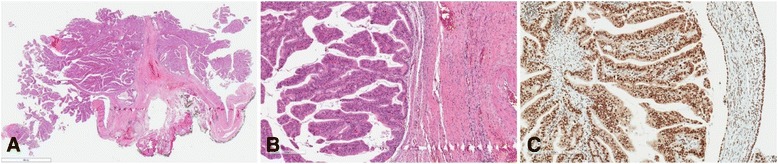
Microscopic examination (hematoxylin and eosin–stained slides). **a** General view of the tumor shows villous pattern of growth (original magnification, ×6). **b** Cytological details of the tumor cells: slight nuclear atypia with no invasive growth (original magnification, ×68). **c** Strong nuclear reaction with MSH2 immunomarker (original magnification, ×98)

Histologically, the tumor consisted of branching papillary structures lined by tall columnar epithelium with slight nuclear atypia and scant fibrous stroma (Fig. 3a, b). Invasive growth was not detected. The adenoma was tested for microsatellite instability using immunostaining of MLH1, PMS2, MSH2, and MSH6 proteins. Positive nuclear immunostaining was observed in tumor cells (Fig. 3c), which showed no evidence of microsatellite instability.

The patient’s postoperative course was uneventful. Her hospitalization period was 10 days. At two visits during the 18-month follow-up period, she had no complications or recurrence.

## Discussion

All types of extrahepatic bile duct benign tumors are very rare compared with malignant tumors. They account for 0.1 % of biliary tract operations and 6 % of all extrahepatic bile duct masses [3].

The World Health Organization classification divides benign epithelial gallbladder and extrahepatic bile duct tumors into tubular, papillary, and tubulopapillary adenomas; biliary cystadenoma; and papillomatosis (adenomatosis) [4]. Tubulovillous adenomas can occur at any site of the gastrointestinal tract but are usually located in the colon and rectum and less frequently in the small bowel. They are extremely rarely found in the CBD [1]. The most common site for villous adenomas in the biliary duct tree is the distal part of the CBD [2, 5]. Villous adenomas in the CBD are histologically similar to those in the ampullary region, gallbladder, or intestine. Several studies have shown that a small proportion of adenomas progress to carcinoma, and there are also general considerations that benign tumors can have similar biological behavior, such as adenoma-to-carcinoma sequences in the colon [4, 6, 7].

Although in general they are rare, benign extrahepatic bile duct tumors occur with greater frequency in the sixth decade of human life [7–9]. Adenomas of extrahepatic bile ducts are usually symptomatic and cause biliary obstruction. Jaundice, intermittent pain, dyspepsia, weight loss, nausea, vomiting, malaise, and fever are common presenting features of benign biliary tumors [3]. Our patient presented with only obstructive jaundice. These tumors rarely grow large enough to become palpable, owing to their anatomical site. Their size usually varies between 1 and 3 cm in diameter [4, 8]. Laboratory studies usually show elevation of blood bilirubin, alkaline phosphatase, and liver enzyme levels [8]. Ultrasound and CT show an intraductal tumor with dilation of the CBD and the intrahepatic biliary tree [2, 3].

Differentiating a villous adenoma from a malignant tumor preoperatively is complex. These tumors have similar clinical presentations [10]. The possibility of invasive carcinoma increases with tumor size and number of lesions [1]. Sonograms, endoscopic ultrasound, CT, and endoscopic retrograde or magnetic resonance cholangiopancreatographies differentiate these tumors with limited success [1, 10]. In imaging-based studies, researchers have found that only 60 % of patients with extrahepatic bile duct adenomas have dilated biliary ducts [3]. According to Stewart *et al.*, brush cytology is a useful technique for initial investigation in patients with suspected pancreaticobiliary neoplasias [11].

Because of complex diagnostics, all previously described factors are evaluated to determine a treatment strategy. Surgical intervention might be appropriate in patients with apparently localized disease, regardless of the cause of the stricture. Patients might have a higher risk of recurrence if they are treated by endoscopic resection alone [12, 13]. Radical resection should be advised if malignancy is suspected or the tumor size is more than 2 cm [7, 13, 14]. Also, pancreaticoduodenectomy should be considered mandatory in cases involving cancer of the distal CBD [7]. Local resection alone might be curative for high-risk patients who are thought to have benign tumors, such as our patient [14, 15]. Also, it is believed that local resection with lymph node dissection of the hepatoduodenal ligament might be curative in cases of malignancies in the midpart of the CBD [14], but this requires further study.

Studies have shown that there are no complications or recurrences after local resection of extrahepatic bile duct adenoma; however, in all of the cases reported in those studies, the follow-up period was less than 1–2 years [8, 14].

## Conclusions

Villous adenoma is a rare extrahepatic biliary tree benign tumor. It is important to diagnose and resect it early because only surgery can rule out the malignancy. A tumor can be found without malignancy even if its size reaches 3 cm or more. Therefore, surgical resection alone may be safe and curative for bulky tumors.

## Consent

Written informed consent was obtained from the patient for publication of this case report and any accompanying images. A copy of the written consent is available for review by the Editor-in-Chief of this journal.

## Abbreviations

ALP: alkaline phosphatase
ALT: alanine transaminase
AST: aspartate transaminase
CBD: common bile duct
CHD: common hepatic duct
CT: computed tomography
DBIL: direct (conjugated) bilirubin
GGT: γ-glutamyl transpeptidase
TBIL: total bilirubin
